# Parental overweight and hypertension are associated with their children’s blood pressure

**DOI:** 10.1186/s12986-019-0357-4

**Published:** 2019-05-27

**Authors:** Renying Xu, Xiaomin Zhang, Yiquan Zhou, Yanping Wan, Xiang Gao

**Affiliations:** 1 Department of Clinical Nutrition, Ren Ji Hospital, School of Medicine, Shanghai Jiao Tong University, Shanghai, China; 2 Department of Nutritional Science, The Pennsylvania State University, University Park, PA 16802 USA

**Keywords:** Parents, Children, Body mass index (BMI), Waist circumference (WC), Percentage of body fat (PBF), Blood pressure

## Abstract

**Background:**

We evaluated the association between parental factors (overweight, history of hypertension, and education level) and children’s blood pressure status. Further, we evaluated to what extent the potential association could be interpreted by children’s adiposity indices.

**Methods:**

The current study included 3316 Chinese school students (1579 girls and 1737 boys, aged 6–14 years) and their parents. Parents reported information on their height, body weight, history of hypertension, and the highest education level. Trained medical staff measured children’s blood pressure, height, body weight, waist circumference (WC), and percentage of body fat (PBF, assessed by bio-impedance method). Z-score of all three indices were calculated and used in the analysis. We used generalized linear model to evaluate the association between parental information and z-score of children’s blood pressure. Meditation analysis was used to evaluate the proportion contributed by z-score of children’s adiposity indices (BMI, WC, and PBF).

**Results:**

We found that parental overweight and hypertension, but not parental education level, were significantly associated with children’s systolic and diastolic blood pressure (*P* < 0.05 for all). Approximately 30.4–92.2% of the association between these two parental factors and children’s systolic blood pressure were mediated by children’s adiposity indices, and 22.3–55.6% for children’s diastolic blood pressure. The strongest meditative factor, among the three obesity indices, was children’s BMI z-score.

**Conclusions:**

The association between parental factors and children’s blood pressure was mainly mediated by children’s adiposity indices.

**Electronic supplementary material:**

The online version of this article (10.1186/s12986-019-0357-4) contains supplementary material, which is available to authorized users.

## Background

The prevalence of hypertension among Chinese children and adolescents increased from 6.9 to 10.7% in the past two decades based on a National survey [1]. Without proper intervention, childhood hypertension will track into adult and dramatically exaggerate adult hypertension [2] and related cardiovascular diseases such as stroke and acute myocardial infarction [3, 4]. Identifying risk factors for childhood hypertension is of significance to ameliorate medical burden, which is associated with adulthood cardiovascular diseases.

Several parental factors, such as obesity [5–7], history of hypertension [8–10], education level [11–13], and family income [11, 12, 14, 15], were shown to be associated with childhood hypertension. It is meaningful to block the transmission from parents to children if we could find the underlying mediators. Previous studies reported that these associations might be mediated by children’s body mass index (BMI), a key driving factor for childhood hypertension [12, 14–16]. However, evidence remains inconsistent: positive [5, 6] and null [7] association between parental BMI and children’s blood pressure were reported in previous studies. Further, whether waist circumference (WC) and percentage of body fat (PBF) in parents, which are believed to be better indices in reflecting body fat than BMI, are associated with offspring blood pressure status remains unclear. Therefore, we performed a cross-sectional study in 3316 Chinese parents-children trios to evaluate the association between parental factors (overweight, history of hypertension, and education level) and childhood blood pressure status. We further evaluated meditative effect of children’s adiposity indices, including BMI, waist circumference (WC), and percentage of body fat (PBF)] on these potential associations.

## Methods

### Study population

The current study was conducted in five primary schools in Gao Hang Town, Shanghai, China, as detailed previously [17]. Parental information including height, body weight, history of hypertension, and the highest education level was self-reported via a questionnaire. Trained medical staff measured children’s blood pressure in September 2014. We have 28 investigators to perform the survey. Among them, 6 doctors and 4 nurses assessed children’s blood pressure, 10 registered dietitians conducted face-to-face interview, and the remain trained medical staffs collected anthropometrical data (e.g., body weight and waist circumference). Approximately 300 children were surveyed during each visit. The number of eligible participants was 3781, after excluding those who declined to participate the study (*n* = 58), those born preterm (*n* = 77), those with missing data (*n* = 325), this resulted in 3321 participants. We further excluded five participants old than 14 years, included were 3316 parents-children trios (1579 girls and 1737 boys, aged 6–14 years) in the analysis. The details of participant recruitment were shown in Additional file 1: Figure S1. Parents signed informed consent forms. The study was approved by the Ethics Committee of Ren Ji Hospital, School of Medicine, Shanghai Jiao Tong University.

### Exposures

Parents reported their information on height, body weight, history of hypertension, and the highest education level via a questionnaire. Parental BMI was calculated and categorized as following: ‘normal’ (BMI < 24.0 kg/m^2^), *or* ‘overweight’ (BMI ≥24.0 kg/m^2^) according to the Working Group of Obesity in China (WGOC) criteria for adults [18]. Parental education level was categorized as ‘low’ (≤middle school) *or* ‘high’ (≥high school) while history of hypertension was categorized as ‘yes’ *or* ‘no’.

### Outcome

Children’s resting blood pressure (BP) was measured by 6 doctors and 4 nurses twice on the children’s right arm in a quiet room after resting for at least 10 min, using a mercury sphygmomanometer and a special cuff that fit 2/3 of the children’s arm. Systolic blood pressure (SBP) is the point at which the onset of Korotkoff sounds and the fourth Korotkoff sound (K4) was used to define diastolic blood pressure (DBP) [19]. The interval between the two blood pressure measurements was at least 10 min and the average value of BP was recorded to the nearest 1 mmHg. Because children’s body size changes rapidly during development and blood pressure keeps changing as well, z-score of blood pressure was calculated based on a national survey [20].

### Potential mediators

We examined whether children’s obesity indices (BMI, WC, and PBF) were mediators for the association between parental information (e.g., obesity status) and childhood blood pressure. Children’s height (to the nearest 0.1 cm), weight (to the nearest 0.1 kg), and body fat (BIA method; TBF-410, Tokyo, Japan) were measured while children were barefoot and in underwear. Body fat was recorded as PBF (fat mass/body weight*100) to the nearest 0.1%. BMI was calculated as the body weight (kg) divided by the height squared (m^2^). BMI z-score was calculated according to the Shanghai age and sex specific height and body weight 2007 standards [21]. WC was measured at the midpoint between the iliac crest and the lower rib (to the nearest 0.1 cm). Z-score of PBF and WC was also calculated [22, 23].

### Assessment of other potential confounders

We collected children’s information on age, sex, birth weight, infant feeding pattern (breastfeeding, part breastfeeding, *or* bottle-feeding), physical activities (< 1 *or* ≥ 1 h per day), and night sleep duration (< 9 *or* ≥ 9 h per day), consumption of carbonated beverage (≤3 *or* ≥ 4 bottles per week), western fast food (≤4 *or* ≥ 5 times per month), traditional Chinese fried food (≤4 *or* ≥ 5 times per week), and processed meat (≤4 *or* ≥ 5 times per week), by the aforementioned questionnaire, which was completed by the parents.

### Statistical analyses

We performed all statistical analyses by SAS version 9.4 (SAS Institute, Inc., Cary, NC). Formal hypothesis testing was two-sided with a significant level of 0.05.

Potential mediator were examined using the method described by Baron and Kenny’s [24]. We used **PROC CORR** to evaluate the association between parental BMI and z-score of children’s adiposity indices and z-score of blood pressure adjusting for children’s age, sex, birth weight, infant feeding, diet, physical activities, and night sleep. **PROC GLM** was used to evaluate the association between parental BMI and other factors (history of hypertension and education level) and children’s blood pressure. We included a number of potential confounders in the model: age (y), sex, height (cm), birth weight (g), infant feeding (breastfeeding, part breastfeeding *or* bottle-feeding), consumption of carbonated beverage (≤3 *or* ≥ 4 bottles per week), western fast food (≤4 *or* ≥ 5 times per month), traditional Chinese fried food (≤4 *or* ≥ 5 times per week), and processed meat (≤4 *or* ≥ 5 times per week), daily physical activities (< 1 h or ≥ 1 h), and night sleep (< 9 *or* ≥ 9 h per day).

To evaluate total effect, indirect effect, and direct effect of the association between parental information and z-score of children’s blood pressure, we performed two GLM regression model: regressing the mediator(s) on the exposures and confounders (as mentioned above), and regressing the outcome on the exposure, mediator(s), and confounders, from which we obtained the indirect effect, direct effect, and the total effect [25]. The meditative proportion was calculated as indirect effect divided by total effect.

## Results

The average paternal and maternal BMI was 24.3 ± 3.0 kg/m^2^ and 21.8 ± 2.9 kg/m^2^ respectively; while average SBP and DBP for children was 101.1 ± 11.3 mmHg and 61.8 ± 8.2 mmHg. The differences in children’s z-score of BMI, WC and PBF were significant between normal-weight and overweight parents (Table 1). Both parental BMI and z-score of all three children’s adiposity indices were associated with z-score of blood pressure in boys and girls (Additional file 1: Table S1).

**Table 1:** Demographic characteristics in 3316 Chinese school students across parental groups

Variable	Group	Father		*P* value	Mother		*P* value
		Normal	overweight		Normal	Overweight	
Sample number	–	2219	1097	–	2892	424	–
Age, y	–	9.5 ± 2.1	8.8 ± 1.9	< 0.01	9.2 ± 2.1	9.1 ± 2.0	0.1
Sex, girls, %	–	48.0	47.0	0.59	47.4	49.3	0.46
Birth weight, g	–	3379 ± 453	3391 ± 458	0.49	3371 ± 447	3465 ± 496	< 0.01
Height, cm	–	141.1 ± 13.7	138.4 ± 12.6	< 0.01	140.0 ± 13.3	139.9 ± 13.4	0.84
Body weight, kg	–	36.0 ± 12.8	35.7 ± 12.5	0.53	35.5 ± 12.5	37.7 ± 13.2	< 0.01
BMI z-score^a^	–	−0.09 (−0.89, 0.74)	0.29 (− 0.48, 1.11)	< 0.01	− 0.01 (− 0.81, 0.84)	0.47 (− 0.29, 1.28)	< 0.01
WC z-score^a^	–	0.18 (− 0.46, 0.92)	0.45 (− 0.21, 1.2)	< 0.01	0.24 (− 0.4, 1.0)	0.61 (− 0.04, 1.38)	< 0.01
PBF z-score^a^	–	− 0.5 (− 1.01, 0.29)	−0.26 (− 0.86, 0.56)	< 0.01	−0.46 (− 0.98, 0.33)	−0.05 (− 0.77, 0.81)	< 0.01
SBP z-score^a^	–	2.32 (0.003, 4.58)	2.76 (0.24, 4.61)	0.17	2.45 (0.13, 4.57)	2.37 (0.21, 4.8)	0.53
DBP z-score^a^	–	−2.38 (−4.48, − 0.26)	−2.63 (− 4.39, − 0.14)	0.16	−2.61 (− 4.46, − 0.25)	−1.9 (− 4.31, − 0.05)	0.02
Infant feeding, %	Breast	50.2	50.7	0.89	50.4	50.5	0.94
	Part breast	19.0	17.2		18.4	18.6	
	Bottle	30.7	32.0		31.2	30.9	
Western fast food, %	≤4 /month	97.8	97.7	0.9	97.8	97.4	0.59
	≥5 /month	2.2	2.3		2.2	2.6	
Traditional Chinese fried food, %	≤4 /week	98.3	99.3	0.03	98.6	98.8	0.73
	≥5/week	1.7	0.7		1.4	1.2	
Processed meat, %,	≤4 /week	97.7	99.0	< 0.01	98.1	98.1	0.98
	≥5 /week	2.3	1.0		1.9	1.9	
Carbonated beverage, %	≤3 /week	90.9	92.2	0.21	91.4	90.6	0.57
	≥4 /week	9.1	7.8		8.6	9.4	
Daily physical activity, %	< 1 h	47.1	32.3	< 0.01	43.5	33.3	< 0.01
	≥1 h	52.9	67.6		56.5	66.7	
Night sleep duration, %	< 9 h	24.8	20.8	0.01	23.5	23.1	0.86
	≥9 h	75.2	79.2		76.5	76.9	
Paternal education level, %	≤ middle school	53.5	54.7	0.51	52.7	61.8	< 0.01
	≥high school	46.5	45.3		47.3	38.2	
Parental history of hypertension, %	No	89.1	72.6	< 0.01	84.1	80.4	0.06
	Yes	10.9	27.4		15.9	19.6	
Maternal education level, %	≤ middle school	56.0	61.1	< 0.01	56.0	68.6	< 0.01
	≥high school	44.0	38.9		44.0	31.4	
Maternal history of hypertension, %	No	96.6	95.8	0.27	97.8	85.9	< 0.01
	Yes	3.4	4.2		2.1	14.1	

*BMI* body mass index, *WC* waist circumference, *PBF* percentage of body fat, *SBP* systolic blood pressure, *DBP* diastolic blood pressure

Parental BMI was categorized into normal (BMI < 24.0 kg/m^2^) and overweight (BMI ≥ 24.0 kg/m^2^) according to the Working Group of Obesity in China (WGOC) criteria for adults

^a^data were shown as median plus quartile range

Children with overweight, or hypertensive parents had higher z-score of blood pressure, compared to those whose parents’ BMI was normal, or without hypertension, respectively. (Tables 2 and 3, model 2). The results were similar when we assessed the association between parental overweight, hypertension, education, and the value of blood pressure (Additional file 1: Table S2). After further adjustment for children’s BMI z-score, the associations lost significance except for a marginal association between paternal history of hypertension and children’s SBP z-score (Tables 2 and 3, model 3). Adjusting z-score of children’s WC and PBF dramatically attenuated the association, but it remained significant except the relationship between maternal overweight, history of hypertension and their children’s SBP z-score (Tables 2 and 3**,** model 4–5). We did not find association between parental education and childhood blood pressure (Tables 2 and 3, model 2).

**Table 2:** Mean difference and standard deviation of z-score of SBP and DBP across paternal groups in 3316 Chinese school students

		Body weight			History of hypertension			Education level		
Variable	Model	Normal	Overweight	*P* value	No	Yes	*P* value	Low	High	*P* value
	Number	2219	1097	–	2773	543	–	1787	1529	–
SBP z-score	Model 1	0 (ref)	0.29 ± 0.1	0.004	0 (ref)	0.24 ± 0.06	< 0.001	0 (ref)	−0.04 ± 0.05	0.35
	Model 2	0 (ref)	0.18 ± 0.05	< 0.001	0 (ref)	0.23 ± 0.06	< 0.001	0 (ref)	−0.04 ± 0.05	0.37
	Model 3	0 (ref)	0.06 ± 0.05	0.23	0 (ref)	0.16 ± 0.05	0.008	0 (ref)	− 0.05 ± 0.05	0.29
	Model 4	0 (ref)	0.09 ± 0.0.05	0.07	0 (ref)	0.16 ± 0.05	0.006	0 (ref)	−0.05 ± 0.05	0.25
	Model 5	0 (ref)	0.1 ± 0.0.05	0.03	0 (ref)	0.15 ± 0.06	0.01	0 (ref)	−0.03 ± 0.05	0.47
DBP z-score	Model 1	0 (ref)	0.17 ± 0.04	< 0.001	0 (ref)	0.2 ± 0.05	< 0.001	0 (ref)	−0.03 ± 0.04	0.38
	Model 2	0 (ref)	0.16 ± 0.04	< 0.001	0 (ref)	0.18 ± 0.05	< 0.001	0 (ref)	−0.04 ± 0.04	0.28
	Model 3	0 (ref)	0.08 ± 0.05	0.11	0 (ref)	0.09 ± 0.06	0.11	0 (ref)	−0.05 ± 0.04	0.22
	Model 4	0 (ref)	0.11 ± 0.04	0.006	0 (ref)	0.14 ± 0.05	0.005	0 (ref)	−0.05 ± 0.04	0.21
	Model 5	0 (ref)	0.11 ± 0.04	0.005	0 (ref)	0.13 ± 0.05	0.01	0 (ref)	−0.04 ± 0.04	0.33

Paternal overweight includes overweight and obese (BMI ≥ 24.0 kg/m^2^). Low education level refers as the highest level ≤ middle school and high as ≥high school. *SBP* systolic blood pressure, *DBP* diastolic blood pressure

Model 1: adjusted for age (y) and sex

Model 2: adjusted for variables in model 1 and further adjusted for height (cm), birth weight (g), infant feeding (breastfeeding, part breastfeeding *or* bottle-feeding), physical activities (< 1 h *or* ≥ 1 h per day), night sleep duration (< 9 h *or* ≥ 9 h per day), consumption of carbonated beverage (≤3 *or* ≥ 4 bottles per week), western fast food (≤4 *or* ≥ 5 times per month), traditional Chinese fried food (≤4 *or* ≥ 5 times per week), and processed meat (≤4 *or* ≥ 5 times per week)

Model 3: adjusted for variables in model 2 and further adjusted z-score of children’s BMI

Model 4: adjusted for variables in model 2 and further adjusted z-score of children’s waist circumference

Model 5: adjusted for variables in model 2 and further adjusted z-score of children’s percentage of body fat

**Table 3:** Mean difference and standard deviation of z-score of SBP and DBP across maternal groups in 3316 Chinese school students

		Body weight			History of hypertension			Education level		
Variable	Model	Normal	Overweight	*P* value	Negative	Positive	*P* value	Low	High	*P* value
	Number	2892	424	–	3194	122	–	1902	1404	–
SBP z-score	Model 1	0 (ref)	0.2 ± 0.07	0.003	0 (ref)	0.31 ± 0.12	0.009	0 (ref)	−0.005 ± 0.05	0.91
	Model 2	0 (ref)	0.2 ± 0.06	< 0.001	0 (ref)	0.3 ± 0.12	0.01	0 (ref)	−0.006 ± 0.05	0.91
	Model 3	0 (ref)	0.01 ± 0.07	0.84	0 (ref)	0.14 ± 0.13	0.27	0 (ref)	−0.005 ± 0.05	0.86
	Model 4	0 (ref)	0.1 ± 0.06	0.11	0 (ref)	0.15 ± 0.12	0.19	0 (ref)	−0.01 ± 0.05	0.79
	Model 5	0 (ref)	0.09 ± 0.06	0.13	0 (ref)	0.13 ± 0.12	0.27	0 (ref)	−0.001 ± 0.05	0.98
DBP z-score	Model 1	0 (ref)	0.21 ± 0.05	< 0.001	0 (ref)	0.31 ± 0.1	0.002	0 (ref)	0.02 ± 0.04	0.69
	Model 2	0 (ref)	0.19 ± 0.05	< 0.001	0 (ref)	0.31 ± 0.1	0.002	0 (ref)	−0.004 ± 0.04	0.91
	Model 3	0 (ref)	0.09 ± 0.06	0.15	0 (ref)	0.19 ± 0.11	0.08	0 (ref)	−0.006 ± 0.04	0.88
	Model 4	0 (ref)	0.14 ± 0.05	0.009	0 (ref)	0.22 ± 0.1	0.03	0 (ref)	−0.006 ± 0.04	0.84
	Model 5	0 (ref)	0.13 ± 0.05	0.02	0 (ref)	0.2 ± 0.1	0.047	0 (ref)	−0.001 ± 0.04	0.97

Paternal overweight includes overweight and obese (BMI ≥ 24.0 kg/m^2^). Low education level refers as the highest level ≤ middle school and high as ≥high school. *SBP* systolic blood pressure, *DBP* diastolic blood pressure

Model 1: adjusted for age (y) and sex

Model 2: adjusted for variables in model 1 and further adjusted for height (cm), birth weight (g), infant feeding (breastfeeding, part breastfeeding ***or*** bottle-feeding), physical activities (< 1 h *or* ≥ 1 h per day), night sleep duration (< 9 h *or* ≥ 9 h per day), consumption of carbonated beverage (≤3 *or* ≥ 4 bottles per week), western fast food (≤4 *or* ≥ 5 times per month), traditional Chinese fried food (≤4 *or* ≥ 5 times per week), and processed meat (≤4 *or* ≥ 5 times per week)

Model 3: adjusted for variables in model 2 and further adjusted z-score of children’s BMI

Model 4: adjusted for variables in model 2 and further adjusted z-score of children’s waist circumference

Model 5: adjusted for variables in model 2 and further adjusted z-score of children’s percentage of body fat

The meditative proportion for the association between paternal overweight and children’s SBP was 68.1, 51.8, and 46.3% for z-score of children’s BMI, WC, and PBF, respectively, and it ranged from 30.7 to 52.4% for the association between paternal overweight and children’s DBP z-score (Table 4). The proportion ranged from 50.7 to 92.2% for maternal overweight and SBP z-score while it was between 29.6 to 55.6% for maternal overweight and DBP z-score (Table 5). It is similar for the association between parental history of hypertension and z-score of children’s blood pressure (Tables 4 and 5). BMI z-score was the strongest mediator among the three adiposity indices. Further classified children into four groups based on parental body weight generated similar results with main analysis (Additional file 1: Table S3). When classified children into four groups based on history of hypertension, the coefficient lost significance (*r* = 0.12, 95% confidence interval: − 0.22, 0.46) when comparing two extreme groups (Additional file 1: Table S4).

**Table 4:** Adjusted meditative effect of z-score of children’s adiposity indices for the association between paternal overweight and history of hypertension, and z-score of children’s blood pressure in 3316 Chinese school students

Mediator	Effect	Overweight		History of hypertension	
		SBP z-score	DBP z-score	SBP z-score	DBP z-score
	Total effect	0.18 (0.08, 0.27)	0.16 (0.08, 0.24)	0.23 (0.12, 0.35)	0.18 (0.08, 0.28)
BMI z-score	Indirect	0.12 (0.09, 0.15)	0.08 (0.06, 0.11)	0.08 (0.04, 0.12)	0.09 (0.06, 0.12)
	Direct	0.06 (−0.03, 0.15)	0.08 (− 0.02, 0.17)	0.16 (0.04, 0.27)	0.09 (− 0.02, 0.21)
	Proportion	68.1%	52.4%	33.8%	48.0%
WC z-score	Indirect	0.09 (0.06, 0.12)	0.05 (0.03, 0.07)	0.07 (0.04, 0.1)	0.04 (0.02, 0.06)
	Direct	0.09 (−0.005, 0.18)	0.11 (0.03, 0.19)	0.16 (0.05, 0.28)	0.14 (0.04, 0.24)
	Proportion	51.1%	31.5%	30.4%	22.3%
PBF z-score	Indirect	0.08 (0.05, 0.1)	0.05 (0.03, 0.07)	0.08 (0.05, 0,12)	0.05 (0.03, 0.08)
	Direct	0.1 (0.01, 0.19)	0.11 (0.03, 0.19)	0.15 (0.04, 0.27)	0.13 (0.03, 0.23)
	Proportion	46.3%	30.7%	35.1%	29.4%

Paternal overweight includes overweight and obese (BMI ≥ 24.0 kg/m^2^). *SBP* systolic blood pressure, *DBP* diastolic blood pressure. *BMI* body mass index, *WC* waist circumference, *PBF* percentage of body fat

Adjusted for age (y) and sex, height (cm), birth weight (g), infant feeding (breastfeeding, part breastfeeding ***or*** bottle-feeding), physical activities (< 1 h *or* ≥ 1 h per day), night sleep duration (< 9 h *or* ≥ 9 h per day), consumption of carbonated beverage (≤3 *or* ≥ 4 bottles per week), western fast food (≤4 *or* ≥ 5 times per month), traditional Chinese fried food (≤4 *or* ≥ 5 times per week), and processed meat (≤4 *or* ≥ 5 times per week)

Proportion of meditative effect = indirect effect/total effect*100%

**Table 5:** Adjusted meditative effect of z-score of three children’s adiposity indices for the association between maternal overweight and history of hypertension, and z-score of children’s blood pressure in 3316 Chinese school students

Mediator	Effect	Overweight		History of hypertension	
		SBP z-score	DBP z-score	SBP z-score	DBP z-score
	Total effect	0.2 (0.08, 0.32)	0.19 (0.09, 0.3)	0.3 (0.06, 0.54)	0.31 (0.11,0.5)
BMI z-score	Indirect	0.19 (0.14, 0.23)	0.11 (0.08, 0.14)	0.16 (0.08, 0.23)	0.11 (0.06, 0.17)
	Direct	0.02 (−0.13, 0.16)	0.09 (− 0.03,0.2)	0.14 (− 0.11, 0.4)	0.19 (− 0.02, 0.4)
	Proportion	92.2%	55.6%	52.0%	37.3%
WC z-score	Indirect	0.1 (0.07, 0.14)	0.06 (0.04, 0.08)	0.15 (0.08, 0.21)	0.08 (0.04, 0.12)
	Direct	0.1 (−0.02, 0.22)	0.14 (0.03, 0.24)	0.15 (−0.08, 0.38)	0.22 (0.03, 0.42)
	Proportion	50.7%	29.6%	49.3%	27.5%
PBF z-score	Indirect	0.11 (0.07, 0.14)	0.07 (0.04, 0.09)	0.17 (0.1, 0.24)	0.11 (0.06, 0.16)
	Direct	0.09 (−0.0.03, 0.21)	0.13 (0.02, 0.23)	0.13 (−0.1, 0.36)	0.2 (0.002, 0.39)
	Proportion	53.8%	35.7%	56.9%	36.0%

Paternal overweight includes overweight and obese (BMI ≥ 24.0 kg/m^2^). *SBP* systolic blood pressure, *DBP* diastolic blood pressure. *BMI* body mass index,*WC* waist circumference, *PBF* percentage of body fat

Adjusted for age (y) and sex, height (cm), birth weight (g), infant feeding (breastfeeding, part breastfeeding *or* bottle-feeding), physical activities (< 1 h *or* ≥ 1 h per day), night sleep duration (< 9 h *or* ≥ 9 h per day), consumption of carbonated beverage (≤3 *or* ≥ 4 bottles per week), western fast food (≤4 *or* ≥ 5 times per month), traditional Chinese fried food (≤4 *or* ≥ 5 times per week), and processed meat (≤4 *or* ≥ 5 times per week)

Proportion of meditative effect = indirect effect/total effect*100%

## Discussions

In this study including 3361 Chinese parents-children trios, we found that parental overweight and hypertension status were associated with children’s blood pressure. However, the association was largely mediated by children’s obesity status. The strengths of the current study included a large sample size, a comprehensible analysis of three adiposity indices, and adjustment for a wide range of potential confounders such as birthweight, diet, physical activities, and sleep duration.

Our study found that parental overweight and history of hypertension were associated with childhood blood pressure. Consisted with our study, a large cross-sectional study (14,400 children, 7–18 years old) reported parental BMI had a weak but significant association with children’s blood pressure [6]. Parental overweight and obesity were also associated with a high likelihood of elevated blood pressure (≥ age- and sex- specific 90th percentile) in their children [6]. In a cohort study included 3864 children with 5 years of follow-up, paternal pre-conceptional BMI was associated with children’s blood pressure at age 5 even after adjusting children’s height and body weight [5]. Contrast to our results, Veena et al. [7] did not find any significant association between parental overweight and children’s SBP in a cohort study including 504 Indian children and their parents after 9.5-year follow-up. However, anthropometric measurements were recorded for mothers at the baseline (≈30 weeks of gestation) and fathers at 5 years after the baseline. The association between parental history of hypertension and children’s blood pressure was also confirmed by previous studies [8–10]. The possible mechanism remains unclear and both genetic and environmental factors could involve [26]. Children’s diet and behavior are generally modelled by their parents, especially in pre-adolescent stage. Parental diet and behavior were reported to be associated with their offspring’s correspondent indices [27, 28]. Epigenetic information (e.g., overweight and hypertension) could print on parental chromosome, which could be inherited by their offspring, thus exerts potential effects on their offspring’s development [29]. Inconsistent with previous study which reported children with two hypertensive parents were in the highest risk of developing childhood hypertension [30], we failed to find significant differences between children with two normal parents and those with two hypertensive parents. One possible interpretation is the small sample size in the latter group (*n* = 50).

Although the differences in ethnicity, statistical method, and when parental information were collected, could explain part of the disparities among the studies, failure to consider children’s adiposity indices could be one of major limitations of previous studies [6, 8–10, 13–15]. Few studies were performed to analyze meditative effects of the association between parental information and children’s blood pressure [11, 12]. A cohort study analyzed 5604 Hongkong adolescents (≈13 years old) and found that adolescent BMI (24%) mediated the association between parental education and adolescent blood pressure, followed by maternal BMI (18%) [12]. Another cohort study performed in 5843 Netherland children and they also found the association between maternal education and children’s blood pressure was mediated by both children’s and maternal pre-conceptional BMI [11]. One cross-sectional study was performed in 2727 Chinese adolescents (12–16 years) to evaluate the association between parental overweight and the risk of childhood metabolic syndrome. The result also showed children’s BMI represented 66.9–72.9% of the effect of parental overweight on potential metabolic syndrome (children with one or two risk components of metabolic syndrome) [16]. As in our study, children’s adiposity indices could explain a large part of the association between parental overweight, history of hypertension, and children’s blood pressure. Including children’s adiposity indices either made the association loss significance or dramatically attenuated the association. The mechanism of mediation remains unclear. As obesity is responsible for most of hypertension [31], thus could explain why adiposity indices (BMI, WC, and PBF) mediated the association between parental factors and childhood blood pressure.

Our study was the first one to compare the differences in meditative effect of the three adiposity indices (BMI z-score, WC, and PBF). Among them, BMI z-score was the strongest one. Consistently, in our previous study, we also found that BMI had a stronger association with hypertension than WC, or PBF in normal-weight children [17]. This suggests that different role of these obesity indices in pathogenesis of childhood obesity. Further studies are warranted to replicate our findings.

Our study had some limitations. First, we did not collect information on salt intake, one of major dietary determinants of hypertension risk. However, estimating salt intake using dietary records or food frequency questionnaire could be inaccurate. It is also not feasible to assess 24 h urinary sodium excretion as a biomarker of salt intake in a large population-based study [32]. Second, children’s information on consumption of carbonated beverages, western fast food, traditional fried Chinese food, and processed meat as well as daily physical activities and night sleep assessed by a parental questionnaire which was subject to many biases (e.g., recall bias). Residual confounding should be taken into consideration. Residual confounding is also of concern. For example, parents’ dietary behavior was not collected, which could have impact on both exposure and outcome in the current study [27]. We did not collect data on sexual maturation. Body size changed rapidly during puberty [33]. However, these data were transferred these into z-score, which might alleviate potential distractions. Finally, both paternal and maternal body weight was self-reported, which might result in misclassification. However, self-reported weight is generally accurate and had high correlation with measured weight [34]. The age and duration of hypertension for parents were not available in the current study. Further studies with deliberate information on parents are needed to warrant the results.

## Conclusions

The association between parental information and children’s blood pressure was mainly mediated by children’s adiposity indices. Thus, paying more attentions to children’s adiposity indices is meaningful to alleviate the effects of parental risk factor on their children health. Longitudinal studies with detailed information on dietary information in different ethnicity are warranted to confirm our findings.

## Additional file

Additional file 1: **Figure S1.** The process of sample recruitment. **Table S1.** Partial correlation between parental BMI, and z-score of children’s BMI, waist circumference, percentage of body fat, and blood pressure in 3 361 Chinese school students. **Table S2.** Mean difference and standard deviation of blood pressure (mmHg) across parental groups in 3316 Chinese school students. **Table S3**. Adjusted meditative effect of z-score of three children’s adiposity indices for the association between parental overweight and z-score of children’s blood pressure in 3,316 Chinese school students. **Table S4**. Adjusted meditative effect of z-score of three children’s adiposity indices for the association between parental history of hypertension and z-score of children’s blood pressure in 3,316 Chinese school students (DOCX 229 kb)

## Abbreviations

BMI: Body mass index
BP: Blood pressure
DBP: Diastolic blood pressure
PBF: Percentage of body fat
SBP: Systolic blood pressure
WC: Waist circumference
