# Supplementary material for: Parental overweight and hypertension are associated with their children’s blood pressure
Source: Nutr Metab (Lond). 2019 May 27;16:35. doi: 10.1186/s12986-019-0357-4 (PMC6537379; doi:10.1186/s12986-019-0357-4)
Supplement: Supplementary file 1 — Figure S1. The process of sample recruitment. Table S1. Partial correlation between parental BMI, and z-score of children’s BMI, waist circumference, percentage of body fat, and blood pressure in 3 361 Chinese school students. Table S2. Mean difference and standard deviation of blood pressure (mmHg) across parental groups in 3316 Chinese school students. Table S3. Adjusted meditative effect of z-score of three children’s adiposity indices for the association between parental overweight and z-score of children’s blood pressure in 3,316 Chinese school students. Table S4. Adjusted meditative effect of z-score of three children’s adiposity indices for the association between parental history of hypertension and z-score of children’s blood pressure in 3,316 Chinese school students (DOCX 229 kb) [file 12986_2019_357_MOESM1_ESM.docx]

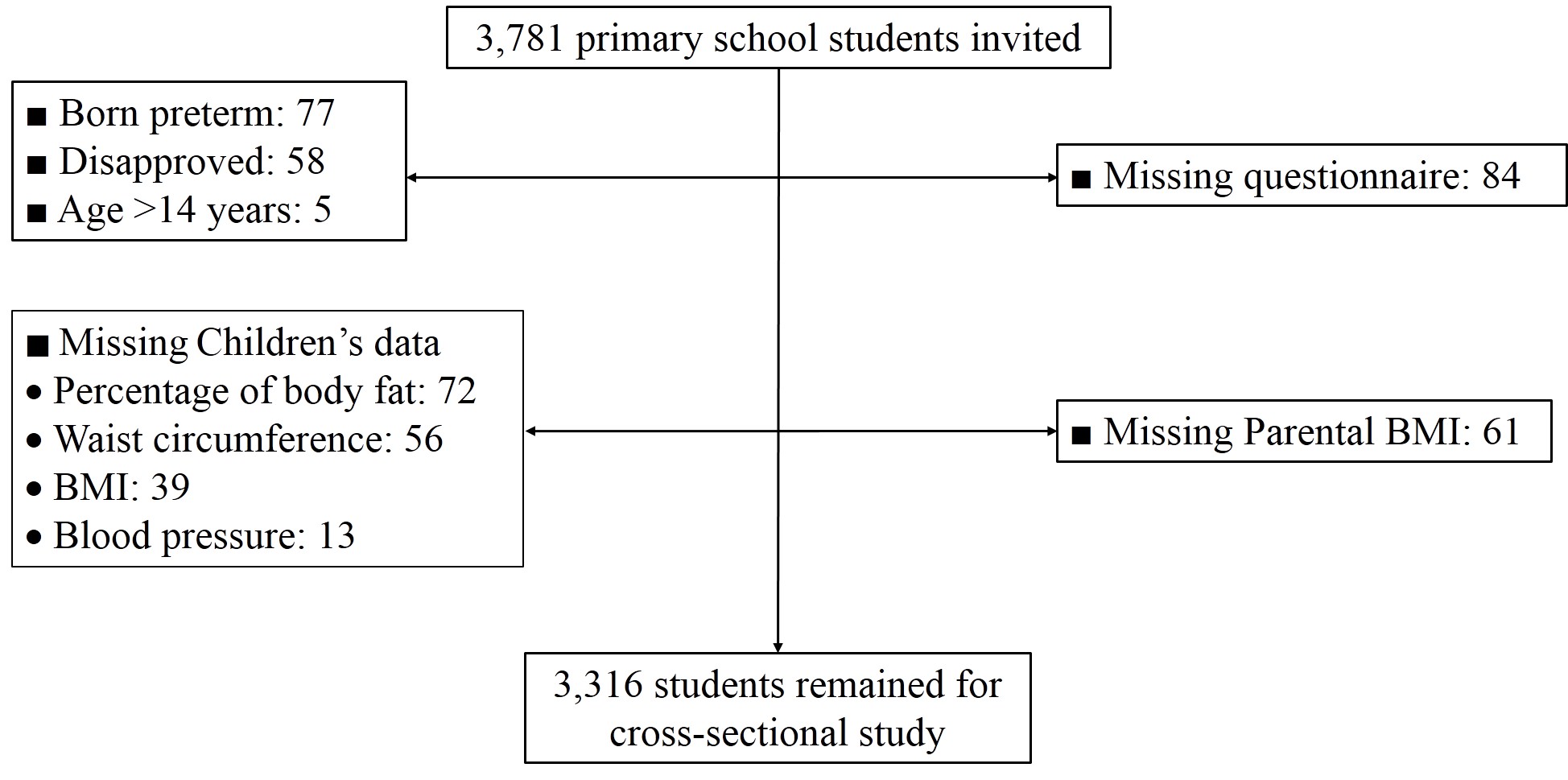


Additional file 1: **Figure S1**. The process of sample recruitment.

Additional file 1: **Table S1**. Partial correlation between parental BMI, and z-score of children’s BMI, waist circumference, percentage of body fat, and blood pressure in 3 361 Chinese school students

| Sex |  | Maternal BMI | BMI z-score | WC z-score | PBF z-score | SBP z-score | DBP z-socre |
| --- | --- | --- | --- | --- | --- | --- | --- |
| Boys | Paternal BMI | r=0.08  p=0.033 | r=0.21  p<0.001 | r=0.19  p<0.001 | r=0.17  p<0.001 | r=0.07  p=0.02 | r=0.06  p=0.03 |
|  | Maternal BMI | -- | r=0.21  p<0.001 | r=0.19  p<0.001 | r=0.17  p<0.001 | r=0.12  p<0.001 | r=0.06  p=0.02 |
|  | BMI z-score | -- | -- | r=0.92  p<0.001 | r=0.8  p<0.001 | r=0.33  p<0.001 | r=0.23  p<0.001 |
|  | WC z-score | -- | -- | -- | r=0.78  p<0.001 | r=0.3  p<0.001 | r=0.19  p<0.001 |
|  | PBF z-score | -- | -- | -- | -- | r=0.25  p<0.001 | r=0.19  p<0.001 |
| Girls | Paternal BMI | r=0.09  p=0.003 | r=0.25  p<0.001 | r=0.22  p<0.001 | r=0.23  p<0.001 | r=0.12  p<0.001 | r=0.11  p<0.001 |
|  | Maternal BMI | -- | r=0.22  p<0.001 | r=0.19  p<0.001 | r=0.22  p<0.001 | r=0.06  p=0.03 | r=0.1  p<0.001 |
|  | BMI z-score | -- | -- | r=0.9  p<0.001 | r=0.94  p<0.001 | r=0.29  p<0.001 | r=0.21  p<0.001 |
|  | WC z-score | -- | -- | -- | r=0.87  p<0.001 | r=0.25  p<0.001 | r=0.17  p<0.001 |
|  | PBF z-score | -- | -- | -- | -- | r=0.3  p<0.001 | r=0.23  p<0.001 |

**Note**: adjusted for children’s age (y), height (cm), birth weight (g), infant feeding (breastfeeding, part breastfeeding ***or*** bottle-feeding), physical activities (<1 hour ***or*** ≥1 hour/day), night sleep duration (<9 hours ***or*** ≥9 hours/day), consumption of carbonated beverage (≤3 ***or*** ≥4 bottles /week), western fast food (≤4 ***or*** ≥5 times /month), traditional Chinese fried food (≤4 ***or*** ≥5 times /week), and processed meat (≤4 ***or*** ≥5 times /week).

Additional file 1: **Table S2.** Mean difference and standard deviation of blood pressure (mmHg) across parental groups in 3,316 Chinese school students

|  |  | Body weight | | | History of hypertension | | | Education level | | |
| --- | --- | --- | --- | --- | --- | --- | --- | --- | --- | --- |
|  | Model | Normal | Overweight | P value | Negative | Positive | P value | Low | High | P value |
| Father | Sample No. | 2,219 | 1097 | -- | 2,773 | 543 | -- | 1,787 | 1,529 | -- |
|  | SBP | **0 (ref)** | 1.72±0.41 | <0.001 | **0 (ref)** | 1.87±0.52 | <0.001 | **0 (ref)** | -0.19±0.41 | 0.63 |
|  | DBP | **0 (ref)** | 1.46±0.32 | <0.001 | **0 (ref)** | 1.42±0.41 | <0.001 | **0 (ref)** | -0.37±0.32 | 0.25 |
| Mother | Sample No. | 2,892 | 424 | -- | 3,194 | 122 | -- | 1,902 | 1,404 | -- |
|  | SBP | **0 (ref)** | 1.9±0.54 | <0.001 | **0 (ref)** | 2.94±1.04 | 0.005 | **0 (ref)** | 0.12±0.42 | 0.78 |
|  | DBP | **0 (ref)** | 1.72±0.42 | <0.001 | **0 (ref)** | 2.8±0.81 | <0.001 | **0 (ref)** | -0.03±0.33 | 0.92 |

**Note**:

1. Parental overweight includes overweight and obese (BMI ≥ 24.0 kg/m^2^). Low education level refers as the highest level ≤middle school and high as ≥high school. SBP, systolic blood pressure; DBP, diastolic blood pressure.

2. Adjusting for age (y), sex, height (cm), birth weight (g), infant feeding (breastfeeding, part breastfeeding ***or*** bottle-feeding), physical activities (<1 hour ***or*** ≥1 hour per day), night sleep time (<9 hours ***or*** ≥9 hours per day), consumption of carbonated beverage (≤3 ***or*** ≥4 bottles per week), western fast food (≤4 ***or*** ≥5 times per month), traditional Chinese fried food (≤4 ***or*** ≥5 times per week), and processed meat (≤4 ***or*** ≥5 times per week).

Additional file 1: **Table S3**. Adjusted meditative effect of z-score of three children’s adiposity indices for the association between parental overweight and z-score of children’s blood pressure in 3,316 Chinese school students

| BP | Mediator | Effect | Parental body weight | | | |
| --- | --- | --- | --- | --- | --- | --- |
|  |  |  | Both of them (-)  (n=1,313) | Father (+) and mother (-)  (n=1,376) | Mother (+) and father (-)  (n=277) | Both of them (+)  (n=350) |
| SBP z-score | -- | Total effect | **Ref** | 0.16 (0.06, 0.25) | 0.26 (0.1, 0.43) | 0.26 (0.11, 0.42) |
|  | BMI z-score | indirect | **Ref** | 0.1 (0.07, 0.13) | 0.13 (0.09, 0.18) | 0.2 (0.15, 0.24) |
|  |  | Direct | **Ref** | 0.05 (-0.05, 0.15) | 0.13 (-0.03, 0.29) | 0.07 (-0.08, 0.22) |
|  | WC z-score | indirect | **Ref** | 0.07 (0.04, 0.09) | 0.09 (0.06, 0.13) | 0.14 (0.1, 0.18) |
|  |  | Direct | **Ref** | 0.09 (-0.01, 0.19) | 0.17 (0.01, 0.33) | 0.13 (-0.02, 0.28) |
|  | PBF z-score | indirect | **Ref** | 0.07 (0.04, 0.09) | 0.09 (0.05, 0.13) | 0.15 (0.11, 0.19) |
|  |  | Direct | **Ref** | 0.09 (-0.01, 0.19) | 0.17 (0.01, 0.33) | 0.12 (-0.04, 0.27) |
| DBP z-score | -- | Total effect | **Ref** | 0.14 (0.05, 0.22) | 0.21 (0.07, 0.35) | 0.24 (0.11, 0.37) |
|  | BMI z-score | indirect | **Ref** | 0.07 (0.05, 0.09) | 0.09 (0.06, 0.12) | 0.13 (0.09, 0.17) |
|  |  | Direct | **Ref** | 0.07 (-0.02, 0.15) | 0.12 (-0.02, 0.26) | 0.11 (-0.02, 0.24) |
|  | WC z-score | indirect | **Ref** | 0.05 (0.03, 0.06) | 0.04 (0.01, 0.06) | 0.12 (0.09, 0.14) |
|  |  | Direct | **Ref** | 0.09 (-0.01, 0.19) | 0.17 (0.01, 0.33) | 0.13 (-0.02, 0.28) |
|  | PBF z-score | indirect | **Ref** | 0.05 (0.03, 0.07) | 0.06 (0.04, 0.1) | 0.11 (0.07, 0.14) |
|  |  | Direct | **Ref** | 0.09 (0.004, 0.17) | 0.14 (0.01, 0.28) | 0.14 (0.01, 0.26) |

**Note**:

1. +, overweight; -, normal.

2. Adjustment for adjusted for age (y) and sex, height (cm), birth weight (g), infant feeding (breastfeeding, part breastfeeding or bottle-feeding), physical activities (<1 hour or ≥1 hour per day), night sleep duration (<9 hours or ≥9 hours per day), consumption of carbonated beverage (≤3 or ≥4 bottles per week), western fast food (≤4 or ≥5 times per month), traditional Chinese fried food (≤4 or ≥5 times per week), and processed meat (≤4 or ≥5 times per week), paternal education level (≤middle school or ≥high school), and maternal education level (≤middle school or ≥high school).

Additional file 1: **Table S4**. Adjusted meditative effect of z-score of three children’s adiposity indices for the association between parental history of hypertension and z-score of children’s blood pressure in 3,316 Chinese school students

| BP | Mediator | Effect | Parental history of hypertension | | | |
| --- | --- | --- | --- | --- | --- | --- |
|  |  |  | Both of them (-)  (n=2,695) | Father (+) and mother (-)  (n=500) | Mother (+) and father (-)  (n=71) | Both of them (+)  (n=50) |
| SBP z-score | -- | Total effect | **Ref** | 0.18 (0.06, 0.31) | 0.32 (0.002, 0.63) | 0.12 (-0.22, 0.46) |
|  | BMI z-score | indirect | **Ref** | 0.04 (0.003, 0.07) | 0.15 (0.06, 0.23) | 0.03 (-0.05, 0.12) |
|  |  | Direct | **Ref** | 0.15 (0.03, 0.27) | 0.17 (-0.14, 0.48) | 0.08 (-0.25, 0.42) |
|  | WC z-score | indirect | **Ref** | 0.03 (0.01, 0.06) | 0.1 (0.03, 0.16) | 0.02 (-0.05, 0.09) |
|  |  | Direct | **Ref** | 0.15 (0.03, 0.27) | 0.22 (-0.09, 0.52) | 0.1 (-0.24, 0.44) |
|  | PBF z-score | indirect | **Ref** | 0.05 (0.02, 0.08) | 0.15 (0.08, 0.22) | 0.02 (-0.05, 0.1) |
|  |  | Direct | **Ref** | 0.13 (0.01, 0.26) | 0.16 (-0.14, 0.47) | 0.1 (-0.24, 0.43) |
| DBP z-score | -- | Total effect | **Ref** | 0.15 (0.04, 0.25) | 0.3 (0.03, 0.56) | 0.18 (-0.11, 0.47) |
|  | BMI z-score | indirect | **Ref** | 0.02 (0.003, 0.05) | 0.1 (0.04, 0.15) | 0.02 (-0.04, 0.08) |
|  |  | Direct | **Ref** | 0.13 (0.02, 0.23) | 0.2 (-0.06, 0.46) | 0.16 (-0.12, 0.44) |
|  | WC z-score | indirect | **Ref** | 0.02 (0.003, 0.04) | 0.06 (0.02, 0.1) | 0.01 (-0.03, 0.06) |
|  |  | Direct | **Ref** | 0.13 (0.03, 0.23) | 0.23 (-0.03, 0.5) | 0.17 (-0.12, 0.45) |
|  | PBF z-score | indirect | **Ref** | 0.03 (0.01, 0.05) | 0.11 (0.05, 0.16) | 0.02 (-0.04, 0.07) |
|  |  | Direct | **Ref** | 0.12 (0.01, 0.22) | 0.19 (-0.07, 0.45) | 0.17 (-0.12, 0.45) |

**Note**:

1. +, positive; -, negative.

2. Adjustment for adjusted for age (y) and sex, height (cm), birth weight (g), infant feeding (breastfeeding, part breastfeeding or bottle-feeding), physical activities (<1 hour or ≥1 hour per day), night sleep duration (<9 hours or ≥9 hours per day), consumption of carbonated beverage (≤3 or ≥4 bottles per week), western fast food (≤4 or ≥5 times per month), traditional Chinese fried food (≤4 or ≥5 times per week), and processed meat (≤4 or ≥5 times per week), paternal body weight (≤24.0 kg/m^2^ or ≥24.0 kg/m^2^), and maternal body weight (≤24.0 kg/m^2^ or ≥24.0 kg/m^2^).
